# Prostate diseases and microbiome in the prostate, gut, and urine

**DOI:** 10.1016/j.prnil.2022.03.004

**Published:** 2022-03-29

**Authors:** Makito Miyake, Yoshihiro Tatsumi, Kenta Ohnishi, Tomomi Fujii, Yasushi Nakai, Nobumichi Tanaka, Kiyohide Fujimoto

**Affiliations:** 1 Department of Urology, Kashihara, Nara, 634-8522, Japan; 2 Department of Diagnostic Pathology, Kashihara, Nara, 634-8522, Japan; 3 Department of Prostate Brachytherapy, Nara Medical University, Nara, 634-8522, Japan

**Keywords:** Microbiome, Prostatic hyperplasia, Prostate cancer, Inflammation, Isoflavones

## Abstract

The microbiome in various organs involves a vast network that plays a key role in the health and wellness of the human body. With recent advances in biological technologies such as high-throughput sequencing, transcriptomics, and metabolomics, it appears that the microbial signature varies dynamically among individuals, creating various roles in metabolism, local and systemic inflammation, and host immunity. Urinary and genital organs, including the prostate, seminal vesicles, and urinary bladder, are reservoirs of several bacterial, viral, and fungal communities. Accumulating evidence has suggested profound roles for the gut, urinary, and intraprostate microbiomes in genitourinary benign and malignant diseases. This review article addresses microbiome-related evidence for three major diseases involved in prostate cancer: chronic prostatitis (CP), benign prostatic hyperplasia (BPH), and prostate cancer (PCa). Symptomatic CP is known as CP/chronic pelvic pain syndrome. CP is one of the most common prostate diseases in young men, accounting for 8% of all men visiting a urologic clinic. Although oral medication is the gold standard therapy for patients with BPH, approximately 13% of men present with clinical progression within 4 years after the initiation of treatment, with 5% requiring surgical intervention. The identification of proinflammatory cytokines and pathogens responsible for the clinical progression of BPH is still underway. Several topics regarding the association between PCa and the microbiome are discussed in this review as follows: i) intraprostatic microbiome and the risk of PCa, ii) gut microbiome and PCa, iii) gut microbiome and the risk of radiation-induced side effects, iv) isoflavone intake and equol-producing intestinal flora on PCa, and v) the inhibitory effect of daidzein and equol on tumor growth and progression of PCa. Further studies are required for a comprehensive understanding between the urogenital microbiome and prostate pathogenesis to facilitate the development of preventive and therapeutic approaches for prostate diseases.

## 1 Introduction

Although bacterial infection had been thought to be pathogenic and harmful to the human body in the past, we are currently aware that microorganisms exist in every organ system of the body in a commensal and mutualistic manner^1^^,^^2^. The microbiome in various organs involves a vast network that plays a key role in the health and wellness of the human body. With recent advances in biological technologies such as high-throughput sequencing, transcriptomics, and metabolomics, it appears that the microbial signature varies dynamically among individuals, creating various roles in metabolism, local and systemic inflammation, and host immunity^3^. Over 10 years ago, the National Institute of Health sought to explore the human microbiome to better understand roles in human health and disorders by launching the Human Microbiome Project^4^. As a great achievement, the largest microbial genome databases for various body sites, including the nares, oral cavity, skin, gastrointestinal tract, breast, and urogenital tract were generated, leading to multiple pieces of evidence on a close link between the microbiome with pathological processes such as aging, atherosclerosis, obesity, diabetes, central nervous system dysfunction, and malignancies^5^, ^6^, ^7^, ^8^. The urinary and genital organs, including the prostate and seminal vesicles are a reservoir of several bacterial, viral, and fungal communities^9^. Accumulated pieces of evidence have suggested profound roles of the gut, urinary, and intra-prostate microbiomes in genitourinary (GU) diseases, such as benign prostate hyperplasia (BPH), chronic prostatitis (CP), prostate cancer (PCa), and other malignancies.

Manipulation of microbiota in GU diseases has just begun to seek and develop clinical applications. This review describes the current advancements and understanding in the field of microbiome research and discusses the close relationship between microbiome signature and prostate diseases, including BPH, CP, and PCa. A better understanding of this relationship will drive further research, which can unveil the initiation and progenitors of these diseases and provide reliable predictors of disease and chemoprevention, as well as novel therapeutic modalities.

## 2 CP and microbiome

CP is one of the most common prostate diseases in young men, with an estimated prevalence of 1.8–8.2%^10^. CP is reported to be responsible for approximately 2 million outpatient clinic visits per year, including 8% of all men who visit a urologic clinic^11^. As chronic pelvic and/or GU pain is a primary component of the CP-caused condition, symptomatic CP is called CP/chronic pelvic pain syndrome (CP/CPPS)^12^. A questionnaire-based cross-sectional study enrolling 20- to 59-year-old men in Estonia demonstrated that a third of men suffering from CP/CPPS considered their own health poor, with more frequently diagnosed renal diseases, BPH, sexually transmitted infections, chronic nervous system diseases, and depression as compared with controls without CP/CPPS^13^. In addition, men with CP reported more frequent cystitis and gynecological inflammations in their partners and more occurrences of prostatitis in their relatives. CP/CPPS can have a strong negative impact on the quality of life, with a remarkable complex of comorbidities, habits, and attitudes.

One of the biggest clinical issues is the challenge faced by many clinicians in diagnosing and treating CP/CPPS effectively owing to poor comprehension. Besides genetic factors and central nervous system imbalance, the host-microbiota of the seminal vesicles, prostate, and gut and the partner's genital microbiota could potentially contribute to the onset of CP/CPPS. Researchers have focused on microorganisms in the seminal fluid, urine, and stool of patients with CP/CPPS. Comparison of the microbiota in the specimens between patients with CP/CPPS and controls differed in various urologic disorders, including CP/CPPS. Table 1 lists the previous studies addressing the possible association between CP/CPPS and the composition of the microbiota^14^, ^15^, ^16^, ^17^, ^18^, ^19^, ^20^, ^21^, ^22^, ^23^, ^24^. Choi et al. investigated the different microbiological etiology of CP in Korean patients between a general hospital and a primary care clinic using bacterial culture and PCR detection of expressed prostatic secretion (EPS) and post-massage urine (VB3). They reported culture-positive patients in the primary care clinic were significantly higher than in the general hospital, but the number of PCR positive patients in the primary care clinic was similar to that in the general hospital^16^. Biological technologies such as high-throughput sequencing, transcriptomics, and metabolomics, have been advancing over time, and the influence of infectious factors in the onset of CP/CPPS continues to be studied. Enterobacteriaceae dominance is the classical concept of the development of CP/CPPS, supplemented with recent data, including the etiological role for some species, as well as microorganisms that are associated with sexually transmitted infections (in particular, Chlamydia trachomatis)^25^^,^^26^. New concepts related to pathological conditions are in the context of microbiota diversity in the affected organs. Mändar et al. investigated profiles of semen microbiomes using the HiSeq2000 sequencing of the ribosomal ribonucleic acid V6 region, demonstrating that the semen of patients with CP/CPPS contained fewer health-supporting lactobacilli and had higher species diversity than that of healthy men^20^. Overall, CP/CPPS has several etiological and potentiating contributions not only from infection and inflammation, but also from central nervous system changes and mental stress, and so on. No singular cause of CP/CPPS has been identified and it is most likely a syndrome with multifactorial factors. All factors play important roles in the crosstalk between the human body and microbiome. Recent research found a significant association between disease severity and the degree of dysbiosis in microbiomes of urine and gut in patients with CP/CPPS as compared to unaffected controls^12^. Stapleton et al. conducted randomized, placebo-controlled phase 2 trial of a *Lactobacillus crispatus* intravaginal suppository probiotic (Lactin-V) for prevention of female recurrent urinary tract infection^27^. A significant reduction in recurrent urinary tract infection for the Lactin-V group suggested that modification of urogenital microbiome by probiotic supplementation potentially prevents symptomatic UTI in males. In the future, the successful work-up of patients with CP/CPPS requires assessment of the microbiota composition on aspects of the pathophysiology of prostate inflammation to develop multidimensional treatment modalities that take into consideration major factors of the syndrome.

**Table 1:** Representative studies for the association between chronic prostatitis/chronic pelvic pain syndrome and microbiome.

The first author (Published year)	Material	No. of patients	Methods Aassays	Major findings (*ex*, specifiic pathogens)	Clinical relevance and interpretation	Reference No.
Ivanov et al. (2009)	Seminal fluid	60 men with CP/CPPS and 48 healthy men from 20 to 35 years-old	Aerobic culture	Enterobacteriaceae, enterococci and *Staphylococcus aureus* were not isolated from the control group. heDCA of staphylococci, coryneforms, Enterobacteriaceae, enterococci and micro-cocci from CP/CPPS group were high anti-complement activity 50.	Important to discriminate between different forms of persistent infection of prostate	14
Ivanov et al. (2010)	Seminal fluid	137 men with CP and 48 healthy men	Phenotyping of coagulase-negative staphylococci	A significantly higher proportion of CP/CPPS strains demonstrated inhibition of lysozyme and platelet microbicidal protein. Bacteria isolated from the control men, strains isolated from men with CPS demonstratedmore intensive inhibition of the bactericidal activity of lysozyme (3.8 ± 0.9 microgram⁄mL vs. 0.7 ± 0.5 microgram⁄mL, p < 0.05).	Identifying these phenotypic characteristics inclinical laboratories would be helpful to differentiate which staphylococcal bacteriospermia case should be treated and which should not.	15
Choi et al. (2013)	Expressed prostatic secretion (EPS) Post-massage urine (VB3)	293 men with CP (105 in a general hospital and 188 in a primary care clinic)	Bacterial culture of EPS or VB3 PCR of EPS or VB3	Routine EPS or VB3 culture detected 12 positive culture of 105 patients (11%) in the general hospital, but 77 positive culture of 188 patients (41%) in the primary care clinic. The PCR diagnosis detected 37 positive PCR of 105 patients (35%) in the general hospital, and 65 positive PCR of 188 patients (35%) in the primary care clinic. The proportions of bacterial CP were 47% and 68% in the general hospital and primary care clinic, respectively. The total portion of bacterial CP was 59%.	Culture-positive patients in the primary care clinic were significantly higher than in the general hospital. The number of PCR positive patients in the primary care clinic was similar to that in the general hospital.	16
Nickel et al. (2015)	Initial and midstream urine (VB1 and VB2) Post-massage urine (VB3)	110 men with CP and 115 controls	Next-generation, culture-independent methodology and Multidisciplinary Approach to the study of Pelvic Pain (MAPP)	Overall species and genus composition differed significantly between CP/CPPS and control participants in VB1 (p = 0.002 species level, p = 0.004 genus level) with *Burkholderia cenocepacia* over represented in CP/CPPS. No significant differences were observed at any level in VB2 or VB3 samples.	Assessment of baseline culture-independent microbiological data from male subjects enrolled in the MAPP Network has identified over representation of B cenocepacia in CP/CPPS.	17
Shoskes et al. (2016)	Fecal DNA	25 men with CP/CPPS and 25 controls	MiSeq sequencing of bacterial specific 16S rRNA capture and Taxonomic and bioinformatic analyses using principal coordinate analysis	Patients with CP/CPPS have significantly less gut microbiome diversity which clusters differently from controls, and robustly lower counts of Prevotella, with separation sufficient to serve as a potential biomarker.	The gut microbiome may serve as disease biomarker and potential therapeutic target in CP/CPPS.	18
Shoskes et al. (2016)	Urine DNA	25 patients with CP/CPPS and 25 controls	MiSeq sequencing of bacterial specific 16S rRNA capture, and taxonomic and functional bioinformatic analyses used principal coordinate analysis	Urinary microbiomes from patients with CP/CPPS have significantly higher diversity which cluster differently from controls, and higher counts of Clostridia compared with controls.	Predicted perturbations of functional pathways suggest metabolite-specific targeted treatment. Several measures of severity and clinical phenotype have significant microbiome differences.	19
Mändar et al. (2017)	Seminal fluid DNA	21 men with CP/CPPS and 46 healthy controls	Sequenced using an Illumina paired-end protocol on HiSeq2000 platform	The most abundant phylum in semen was Firmicutes. The counts of lactobacilli were higher in controls than CP/CPPS patients, especially for Lactobacillus iners. Proteobacteria comprised higher proportions in CP/CPPS patients than controls. The species richness was higher in CP/CPPS patients than controls.	The seminal fluid of CP/CPPS patients contains fewer health-supporting lactobacilli, and has higher species diversity than that of healthy controls.	20
Choi et al. (2020)	Seminal fluid	17 men diagnosed with CP/CPPS and four healthy volunteers	Bacterial culture and DNA pyrosequencing	None of the semen samples showed colony formation in conventional bacterial cultures. Pyrosequencing revealed multiple bacterial genera in all samples, including an abundance of fastidious bacteria. Corynebacterium, Pseudomonas, Sphingomonas, Staphylococcus, and Streptococcus were frequently detected nonspecifically in both the patient and control groups. However, Achromobacter, Stenotrophomonas, and Brevibacillus were more frequently found in the CP/CPPS patients.	The identification of dominant species in the CP/CPPS group other than those reported in previous studies might be helpful for future etiological analysis of CP/CPPS.	21
Wu et al. (2020)	Urethral secretions Expressed prostatic secretion (EPS)	33 men with CP/CPPS III and 30 healthy men	Next-generation sequencing	The microbial compositions of the urethral secretions and EPS collected from the same subject were essentially the same.	Changes in the urinary tract microbiome may disrupt the microecological balance of the urinary system, leading to inflammation. Conversely, the true pathogens of CP/CPPS may not be prokaryotic or eukaryotic microorganisms.	22
Suárez et al. (2021)	Urine Seminal fluid	5 men with CP/CPPS 5 and fertile volunteers	Sequencing and Nitric oxide levels and proinflammatory cytokines in seminal and serum	The microbiota present in the semen and urine samples of fertile men presents more operational taxonomical units. Less microbial diversity could be associated with chronic prostatitis symptoms.	CP does not seem to affect male fertility.	23
Kogan et al. (2021)	Post-massage urine (VB3)	170 with a history of CP/CPPS from 18 to 45 years-old	Meares-Stamey test	In patients with CP/CPPS, a predominance of anaerobes or a combination of aerobes and anaerobes in a titer of ≥ 10^3^ colony-forming units per mL in post-massage urine is associated with worse clinical status.	The main components that determine the severity of symptoms are an increase in microbial load and qualitative differences in the composition of the microbiota.	24

CP/CPPS, chronic prostatitis/chronic pelvic pain syndrome; CP, Chronic prostatitis.

## 3 BPH, male lower urinary tract symptoms (LUTS), and the microbiome

Oral medications, such as alpha-1 blockers and/or 5-alpha-reductase inhibitors (finasteride or dutasteride), are gold standard therapies and decrease the risk of clinical progression^28^. Unfortunately, approximately 13% of males present with clinical progression within 4 years after the initiation of treatment, with 5% requiring surgical intervention, such as transurethral resection of the prostate^29^. The fact that some patients show a lack of response or durability to oral medication drives researchers to fully understand the underlying pathophysiology of BPH and benign prostatic enlargement. Although a previous study revealed that an individual's urinary microbiome/microbiota could provide information about bacteria related to urinary urgency incontinence status and treatment response to anticholinergic drugs, there is a lack of evidence regarding predictive tools for treatment response to drugs in patients with BPH^30^. As intraprostatic inflammation may play a vital role in the pathogenesis of BPH and clinical progression,^31^^,^^32^ this physiological phenomenon is a potential target for diagnosis and treatment. Profiling the urinary microbiome in men with positive vs. negative biopsies for PCa led to the hypothesis of a link between the prostate and proinflammatory bacterial species^33^.

An investigation into the association between the microbiota and CP/CPPS paved the way to studying similar associations between the microbiota in BPH and male LUTS (Table 2)^34^, ^35^, ^36^, ^37^, ^38^. A previous study published in 2013 demonstrated that aging affects the microbiota in male midstream voided urine and showed an interesting trend. The total number of bacteria in the distal urethra significantly decreased with age, while the number of genera increased (increased ‘diversity’)^39^. This finding suggested that the change in the urethra and bladder microbiota with age might be associated with increasing LUTS in older males, which is typically due to BPH. Midstream urine, catheterized urine, seminal fluid, expressed prostatic secretion, stool, and resected prostatic tissue have been used for profiling the microbiome. However, Bajic et al. pointed out that voided urine cannot adequately characterize the male bladder microbiome due to the existence of distinct microbiota in the anterior urethra of the male lower urinary tract^35^. In the analysis of urine microbiome from males with and without prostatic enlargement, the International Prostate Symptom Score severity was associated with detectable bacteria in catheterized urine. Moreover, Yu et al. reported the significant difference in the microbiota among voided urine, seminal fluid, and expressed prostatic secretions^34^. Given this evidence, materials used for microbiota profiling surely affect the study findings. Infection with certain bacteria may induce a chronic inflammatory state in the prostate via the enhanced production of proinflammatory cytokines. Further investigation into the urogenital microbiota may reveal potential associations between BPH, male LUTS, and the causative pathogens.

**Table 2:** Representative studies for the association between benign prostatic hyperplasia/lower urinary tract symptoms and microbiome.

The first author (Published year)	Material	No. of patients	Methods Aassays	Major findings (ex, specifiic pathogens)	Clinical relevance and interpretation	Reference No.
Yu et al. (2014)	Urine, seminal fluid expressed prostatic secretion (EPS)	21 men with BPH and 13 men with PCa	16S rRNA gene sequencing with PCR-DGGE analysis	Bacterial flora in expressed prostatic secretions of patients with BPH differ from those with PCa.	Significant changes in the microbial population in EPS, urine and seminal fluid of subjects with prostate cancer and BPH, indicating a possible role for these bacteria in BPH and PCa	34
Bajic et al. (2018)	Midstream voided urine, Catheterized urine	49 men with BPH	EQUC, 16S rRNA gene sequencing	Increased symptom score severity associated with detectable bacteria on catheterized urine, voided urine is inadequate to sample the bladder microbiome.	Voided urine does not adequately characterize the male bladder microbiome. In males with and without BPE, IPSS severity was associated with detectable bacteria in catheterized urine.	35
Holland et al. (2019)	DNA from urine and fecal samples	30 men with LUTS	16S ribosomal RNA gene high-throughput next-generation sequencing platform. The microbial profiles for taxonomy examining the correlation between the different operational taxonomy units (OTUs).	The most substantial negative correlation was between Lachnospiraceae Blautia, a bacteria that increases the availability of gut anxiolytic and antidepressant short-chain fatty acids, and bother. The abundance of L. Blautia continued to have a protective correlation against LUTS when looking at the different levels of IPSS severity.	Ten unique urinary OTUs showed significant correlation with LUTS. No fecal OUT had more than a low correlation with the outcomes of interest.	36
Jain et al. (2020)	DNA and sections from resected tissue	36 men with BPH	Culture and/or next-generation sequencing Immunohistological evaluation of tissue sections	Microbial culture of tissue samples showed the presence of live bacteria in 55.5% of the patient tissues. Majority of the isolates were CPS, E. coli and Micrococcus spp. the presence of multiple bacteria and the most common phylum in the BPH tissues were found to be Proteobacteria, Actinobacteria, Firmicutes, and Bacteroidetes. staining confirmed the presence of cells with damaged DNA lesion in BPH tissues and also correlated with the severity of inflammation.	BPH tissues do have a divergent microbial composition including the commonly found E. coli and these bacteria might contribute to the BPH-associated inflammation and/or tissue damage. The BPH-associated E. coli induced NF-κB signaling and DNA damage in prostate epithelial cells in vitro.	37
Lee et al. (2021)	Midstream voided urine DNA	77 men with BPH and 30 controls	16S Metagenomic Sequencing. Operational taxonomic unit (OTU) clusters are obtained.	Some of bacterial genera present in the samples of the BPH group. Some of bacterial genera correlated with a high IPSS, and severe storage and voiding symptoms.	Dysbiosis of urine microbiota may be related to the development of BPH and the severity of LUTS.	38

BPH, benign prostatic hyperplasia; IPSS, International Prostate Symptom Score; LUTS, lower urinary tract symptoms; PCa, prostate cancer.

## 4 PCa and the microbiome

### 4.1 Intraprostatic microbiome and the risk of PCa

Carcinogenesis is associated with multiple factors, including age, race, diet, heredity, the environment, and inflammation. Approximately one-fifth of malignancies can be attributed to infection by bacteria, viruses, and parasites^40^. Ravich et al. first advocated the infection hypothesis for PCa and the potential of prophylaxis in 1951^41^. Then, a meta-analysis of thousands of patients with PCa and controls from 29 case-control studies showed a significant association between carcinogenesis risk and infection history of any sexually transmitted infection, including Mycoplasma genitalium (MG) and human papillomavirus (HPV)^9^^,^^40^. There has been great interest in understanding the key role of inflammation in the initiation and progression of PCa. Proliferative inflammatory atrophy is a putative pathological lesion wherein inflammation stress drives prostate carcinogenesis through excessive reactive oxygen species, epigenetic alterations, and subsequent mutagenesis^42^. Intense exposure of the numerous microorganisms in the prostate through the urethra can generate chronic inflammation, in which stress drives prostate carcinogenesis through excessive reactive oxygen species, epigenetic alterations, and subsequent mutagenesis. Cavarretta et al. investigated the microbiome of the prostate microenvironment using 16s rRNA gene amplification followed by massive sequencing to identify the specific microbiota or bacteria associated with prostate carcinogenesis^43^. The evidence from this study was limited because there was a lack of evaluation of intraprostatic inflammation such as proliferative inflammatory atrophy and the detection of non-bacterial microorganisms such as mycoplasmas and viruses.

**Figure 1:**
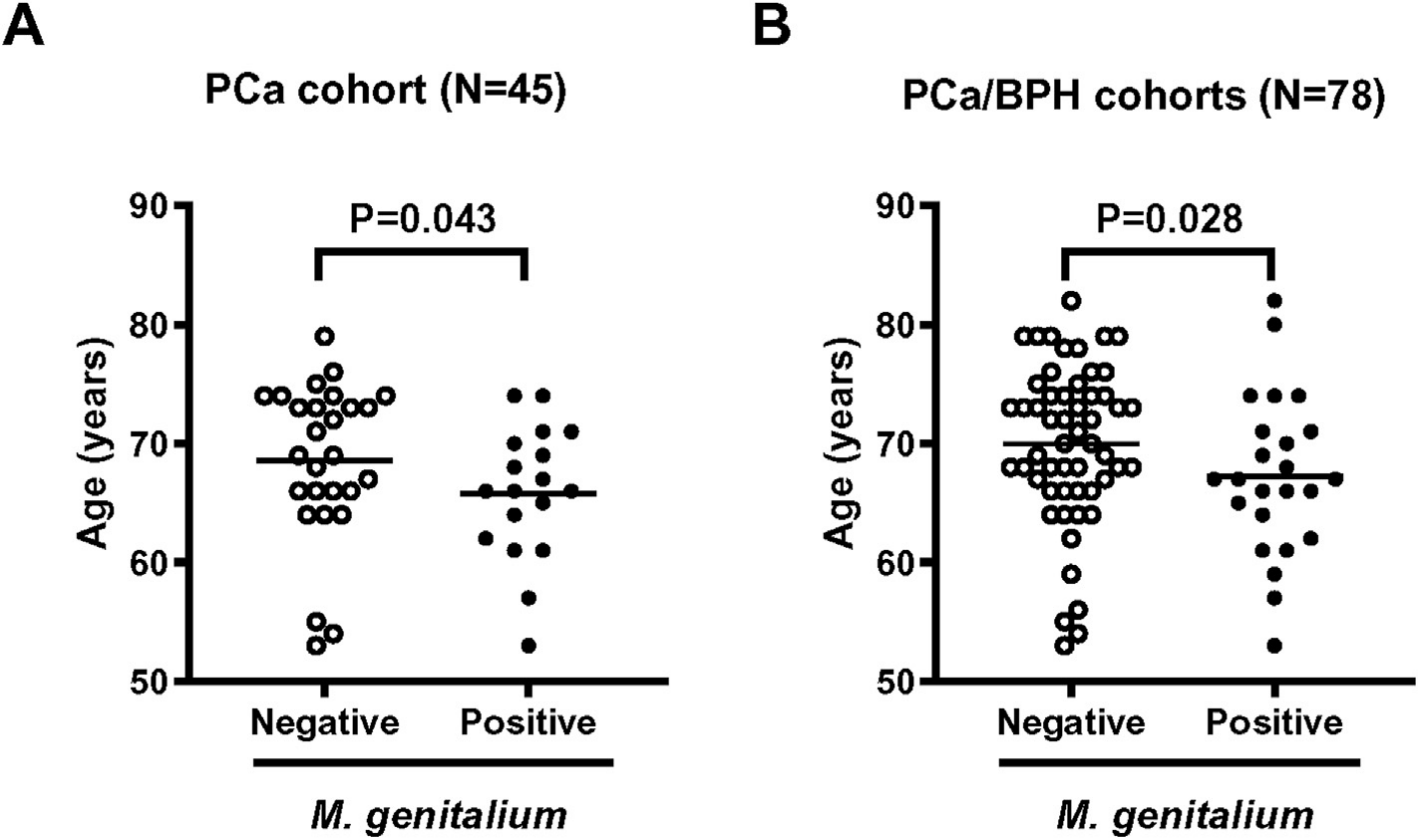
**Association between age and the *Mycoplasma genitalium* infection status in surgical specimens.** Data for patient age at surgery for the *Mycoplasma genitalium*-positive and *Mycoplasma genitalium*-negative groups are shown as scatterplots in the PCa cohort (A) and the PCa/BPH cohort (B) analysis. The Mann–Whitney U test was used to compare the two groups. This figure is cited from Reference number 9. PCa, prostate cancer; BPH, benign prostatic hyperplasia; M. genitalium, *Mycoplasma genitalium*.

We previously examined robot-assisted prostatectomy specimens from 45 patients with PCa and transurethral resection specimens from 33 patients with BPH^9^. Using these specimens and DNA-based detection analysis, we screened tissue infection of a panel of seven sexually transmitted infection-related organisms and compared the pathological severity of prostatic inflammation to seek the potential association of infection, inflammatory environment, and carcinogenesis. Among the tested microorganisms, *Neisseria gonorrhoeae*, *Chlamydia trachomatis*, *Ureaplasma urealyticum*, and *Mycoplasma hyorhinis* were not detected in any specimen. The positive rate of MG was significantly different between the PCa cohort (18/45, 40%) and the BPH cohort (6/33, 18%). HPV18 DNA was detected in five (11%) patients in the PCa cohort and two (6%) patients in the BPH cohort, while only one patient in the PCa cohort was positive for HPV16. Of the six patients with PCa positive for HPV16/18, four (67%) were positive for MG, indicating a high rate of mixed infection. The positive status of MG in surgical specimens was associated with lower age in the PCa cohort (N = 45, Fig. 1A) and the PCa/BPH cohort (N = 78, Fig. 1B). MG is one of the most significant sexually transmitted pathogens, which can cause several human inflammatory diseases such as urethritis and CP in men and cervicitis, endometritis, salpingitis, tubal factor infertility, and pelvic inflammatory disease in women^44^. The higher rate of MG infection in the younger population could be explained by higher sexual activity. In the analysis of MG infection, patient background, and intraprostatic inflammation in the PCa cohort, the rate of extensive disease (pT2c–3b) was higher in MG-positive patients than in MG-negative patients (77% vs. 44%, P = 0.027). However, no significant correlation was observed between the MG infection status and the grade of pathological intraprostatic inflammation.

The possible clinical application of testing prostatic microorganisms would be an indication or recommendation for re-biopsy or close follow-up after negative results of the initial prostate biopsy. We examined the feasibility of detecting infectious agents from the corresponding prostate needle biopsy cores. The detection sensitivity of biopsy specimens was 61% for MG and 60% for HPV18^9^. Although the detection accuracy would be high enough, non-invasiveness of material sampling is important for routine clinical testing. Yu et al. analyzed the feasibility of detecting microorganisms using biological fluids, including urine, expressed prostatic secretion, and seminal fluid samples from patients with PCa or BPH^34^. The investigators successfully detected the presence of diverse bacteria from all three materials and found a significant difference in the microbial population between PCa and BPH. Given that data obtained from non-invasive sampling accurately reflects the microbial environment in the prostate, another feasible and possible intervention would be the targeting of infectious agents by antibiotics to reduce the risk of PCa.

### 4.2 Gut microbiome and PCa

Another topic to be discussed is the close association between gut microbiota and the risk of PCa. Emerging data have proven that the gut microbiota of men with PCa is different from that of men without PCa^45^. Due to racial differences and heterogeneity in the microbiome among patients, its role in prostate carcinogenesis remains unclear, especially in Asian men. Several comparative analyses using stool samples or rectal swabs proved the compositional differences in bacteria between patients with PCa and healthy controls (Table 3)^46^, ^47^, ^48^, ^49^, ^50^, ^51^. Liss et al. collected rectal swabs of men prior to prostate needle biopsies and evaluated the association between the microbiome and PCa status^46^. Bacteroides and streptococcal species were detected as the most abundant bacteria in patients with PCa. Additionally, pathways related to folate and arginine metabolism were altered in patients with PCa. Similarly, Golombos et al. revealed enrichment of Bacteroides in PCa and Faecalibacterium and Eubacterium in non-cancer controls.^47^ Sfanos et al. evaluated rectal swabs from 21 patients with PCa and demonstrated that *Ruminococcaceae* and *Akkermansia muciniphila* were more abundant in men taking oral androgen receptor axis-targeted therapies such as abiraterone acetate and enzalutamide^48^. A Japanese report found that *Rikenellaceae*, *Alistipes*, and *Lachnospira* (all short-chain fatty acid-producing bacteria) were significantly higher in those at a high risk (grade group ≥2) of PCa^50^. The same group previously reported that a high-fat diet and obesity promoted local prostate inflammation and cancer proliferation and that short-chain fatty acids and major metabolites of intestinal bacteria promoted PCa growth via the insulin growth factor-1 signaling pathway in a preclinical model.^52^ Interestingly, compositional differences of the gut microbiome in matched hormone-sensitive and castration-resistant PCa were reported by Liu et al^51^. The abundance of several bacterial florae, including genus *Phascolarctobacterium* and *Ruminococcus*, increased in castration-resistant PCa. Functional analyses revealed that bacterial gene pathways involved in terpenoid/polyketide metabolism and ether lipid metabolism are activated significantly in the transition from hormone-sensitive PCa to castration-resistant PCa.

**Table 3:** Representative studies investigating compositional differences of bacteria in stool samples or rectal swab of patients with prostate cancer.

The first author (Published year)	Material	No. of patients	Methods Aassays	Major findings (ex, specifiic pathogens)	Clinical relevance and interpretation	Reference No.
Liss et al. (2018)	Rectal swabs before prostate biopsy	64 men with PCa and 41 men without cancer	16S rRNA gene sequencing with Communities by Reconstruction of Unobserved States (PICRUSt)	Higher abundance in PCa: Bacteroides, Streptococcus	The authors formed a novel microbiome-derived risk factor for prostate cancer based on 10 aberrant metabolic pathways (area under curve = 0.64, p = 0.02).	46
Golombos et al. (2018)	Stool samples before prostate biopsy	20 men with intermediate or high risk localized PCa 8 men without cancer	Computational genomics analysis using MetaPhlAn2 and HUMAnN2 platforms.	Higher abundance in PCa: Bacteroides massiliensis Higher abundance in controls: Faecalibacterium prausnitzii, Eubacterium rectalie	Biologically significant differences exist in the gut microbial composition of men with PCa and healthy controls.	47
Sfanos et al. (2018)	Rectal swabs from patients with different clinical states of PCa	30 men with PCa or without cancer	16S rDNA amplicon sequencing Functional inference of identified taxa using PICRUSt	Higher in men receiving androgen axis target therapy: Akkermansia muciniphila, Ruminococcaceae spp., Lachnospiraceae spp. Lower in men receiving androgen deprivation therapy: (family) Brevibacteriaceae, Erysipelorichaceae, Streptococcaceae	The authors speculate that oral hormonal therapies may change the gut microbiota, influence clinical responses to therapy, and/or potentially modulate the antitumor effects of future therapies such as immunotherapy.	48
Alanee et al. (2019)	Rectal swabs before prostate biopsy	30 men with PCa and 16 men without cancer	16S rRNA gene high-throughput next-generation sequencing platform Differential analysis of the operational taxonomical units (OTUs)	Higher abundance in PCa: Bacteroides	Analysis of the bacterial taxonomies revealed no clustering in concordance with benign or malignant prostate biopsies.	49
Matsushita et al. (2021)	Rectal swabs before prostate biopsy	96 men with PCa and 56 men without cancer	16S rDNA amplicon sequencing	Higher in high-risk PCa (grade group 2 or higher) group: Rikenellaceae, Alistipes, and Lachnospira (all short-chain fatty acid-producing bacteria	The specific bacterial taxa are associated with high-risk PCa. The gut microbiota profile can be a novel tool for the detection of high-risk PCa.	50
Liu et al. (2020)	Stool samples before and after castration-resistance	21 men with PCa treated with ADT	16S rRNA gene amplicon sequencing Differences in microbiota were determined with α/β-diversity and LefSe analysis. Functional inference of microbiota was performed with PICRUSt.	Increaced abundance in castration-resistant PCa: Phascolarctobacterium and Ruminococcus.	Analysis of the bacterial taxonomies revealed no clustering in concordance with benign or malignant prostate biopsies.	51

PCa, prostate cancer; ADT, androgen deprivation therapy.

### 4.3 Gut microbiome and the risk of radiation-induced side effects

Radiation therapy is one of the gold standard treatments for the broad spectrum of PCa. The biggest concern is radiation-induced side effects such as gastrointestinal toxicity and GU toxicity, which can limit the radiation dose^53^, ^54^, ^55^. The Microbiota- and Radiotherapy-Induced Gastrointestinal Side-Effects study hypothesized that the microbiome differs between patients with and without radiation enteropathy^56^. The results clearly showed that microbiota diversity dynamically decreased over time in patients with increasing radiation enteropathy. Similarly, a consistent relationship between low diversity and late radiation enteropathy has been noted. Metataxonomic (16S rRNA gene) and imputed metataxonomic (Piphillin) analyses demonstrated that a higher abundance of *Clostridium IV*, *Roseburia*, and *Phascolarctobacterium* were associated with radiation enteropathy. Homeostatic intestinal mucosal cytokines linked with microbiota regulation and maintenance of the intestinal wall were decreased in radiation enteropathy. Moreover, interleukin-15 levels were inversely correlated with the counts of *Roseburia* and *Propionibacterium*. Microbiome testing before and during pelvic radiation would exhibit the potential for risk assessment, prevention, or treatment of radiation-induced enteropathy.

### 4.4 Isoflavone intake and equol-producing intestinal flora on the risk of PCa

The age-adjusted incidence of PCa is lower in Asian men than that in Western populations. According to the 2020 worldwide cancer statistics, the incidence rates of PCa vary from 6.3 to 83.4 per 100,000 men across regions, with the highest rates found in Northern and Western Europe, the Caribbean, Australia/New Zealand, Northern America, and Southern Africa and the lowest rates in Asian countries^57^. Moreover, the statistic of region-specific mortality age-standardized rates for PCa in 2020 shows lower mortality rates in Asian countries^57^. The A substantial number of studies have assessed dietary habits in association with PCa risk. One of the most attractive foods has been products from soybean, a traditional Japanese meal, or isoflavones, which may be associated with a decreased risk of incidence and progression of PCa^58^. A large epidemiological survey of multiethnic men in Hawaii and Los Angeles demonstrated that men with the highest intake of legumes lowered the risk of PCa by 11% and the risk of nonlocalized or high-grade PCa by 26% compared to men with the lowest intake of legumes^59^. Separate analyses showed that similar risk reductions were noted for soy products and legumes; however, the isoflavones in soy products were probably not responsible for this effect. The main mechanism of the inhibitory effect against PCa is that isoflavones function in the human body as strong phytoestrogens.

The three most abundant isoflavones are daidzein, genistein, and glycitein. The former two together comprise 0.1–0.3 mg/g soybean^60^. Moreover, daidzein is converted by a specific human intestinal bacterium, *Slackia sp.* NATTS, to equol, which is biologically more active than any other isoflavones^60^^,^^61^. Equol exerts a strong antiandrogen function by binding to dihydrotestosterone (DHT)^62^. A person who can produce equol in response to consuming soy isoflavones is classified as an equol producer. It is estimated that health benefits from soy-based diets may be greater in equol producers than in equol non-producers^58^^,^^63^. We previously evaluated the effect of supplementing healthy men with soy isoflavones on the serum levels of sex hormones, which were involved in PCa development^64^. A total of 28 healthy Japanese volunteers (age, 30–59 years), comprising 18 equol producers and 10 equol non-producers, were given soy isoflavone (60 mg daily) supplements for 3 months, and their sex hormone levels were at the baseline and after 3 months were compared. No changes in the serum levels of estradiol and total testosterone were observed, while a significant increase in the serum levels of sex hormone-binding globulin and a significant decrease in the serum levels of free testosterone and DHT after 3 months of supplementation were detected. Interestingly, in two out of 10 equol non-producers, equol in the serum was detectable after 3 months of supplementation. Our findings demonstrated that short-term supplementation with soy isoflavones stimulated the production of serum equol and decreased the serum DHT level in healthy Japanese volunteers, leading to a possible PCa risk reduction. Another by-product of our study was the possibility of converting equol non-producers to equol producers by prolonged and consistent intake of soy isoflavones.

Epidemiological evidence of serum isoflavone levels and equol production by a specific human intestinal bacterium has long been evaluated with regard to the risk of PCa. A Japanese case-control study collecting data on serum isoflavone levels, NATTS identification in feces, and semi-quantitative frequency of food intake from patients with PCa and controls was completed and reported in 2016^65^. Higher serum levels of genistein, daidzein, and glycitein were significantly associated with a decreased risk of PCa, while serum levels of equol and NATTS in feces were not associated. A meta-analysis of two studies from Japan and five studies from Europe summarized the data of prediagnostic circulating levels of isoflavones (genistein, daidzein, and equol) and lignans (enterolactone and enterodiol) and the risk of PCa from 2828 patients with PCa and 5593 controls.^66^ Overall, there is no strong evidence regarding the association between prediagnostic circulating levels of isoflavones or lignans and the risk of PCa. However, the authors emphasized the need for further research in populations where isoflavone intake is high, as in Japan, because of the positive link between higher circulating equol and a lower risk of PCa.

### 4.5 Inhibitory effect of daidzein and equol on tumor growth and progression of PCa

According to an epidemiological study, isoflavone consumption was associated with a lower incidence of metastatic PCa,^67^ supporting experimental data showing the potential inhibitory effect of isoflavones against the metastatic capability of tumor cells^68^. The molecular mechanism underlying this inhibitory effect is still unclear. Therefore, we focused on the possible inhibitory effect of isoflavones on epithelial-mesenchymal transition (EMT) in PCa. EMT has been implicated in the progression and metastasis of various malignant tumors, including PCa^69^^,^^70^.

**Figure 2:**
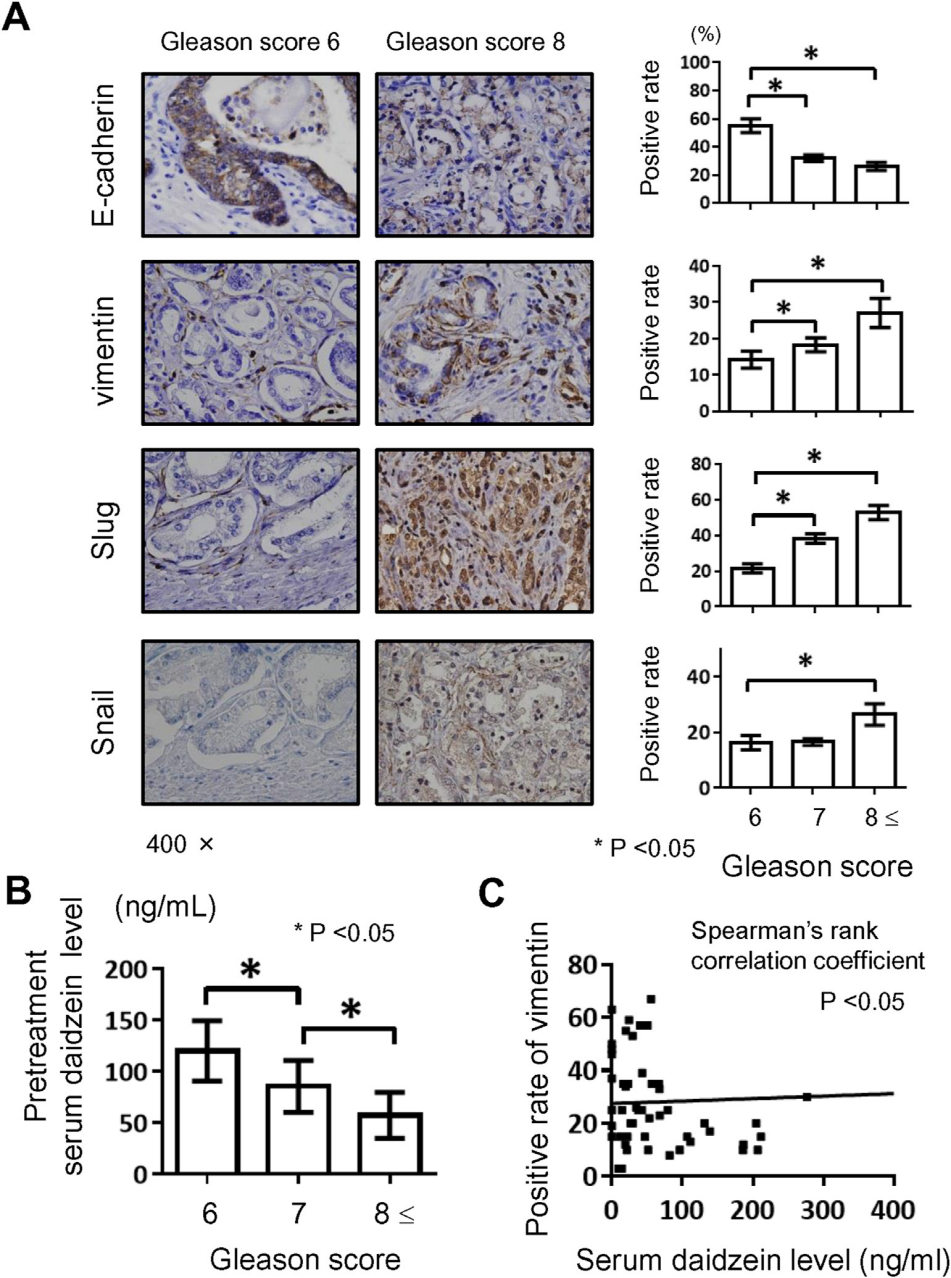
**Association between epithelial-mesenchymal transition, high Gleason’ score, and pretreatment serum daidzein level in patients with localized prostate cancer.** A) Immunohistochemical staining analysis using primary antibodies against E-cadherin, vimentin, Slug, and Snail. The association between each marker and the Gleason grade are evaluated. B) Pretreatment serum daidzein level among tumors with different Gleason scores is compared using the Mann–Whitney U test. C) Correlation between the pretreatment serum daidzein level and the rate of vimentin-positive cancer cells are tested using Spearman's rank correlation coefficient.

**Figure 3:**
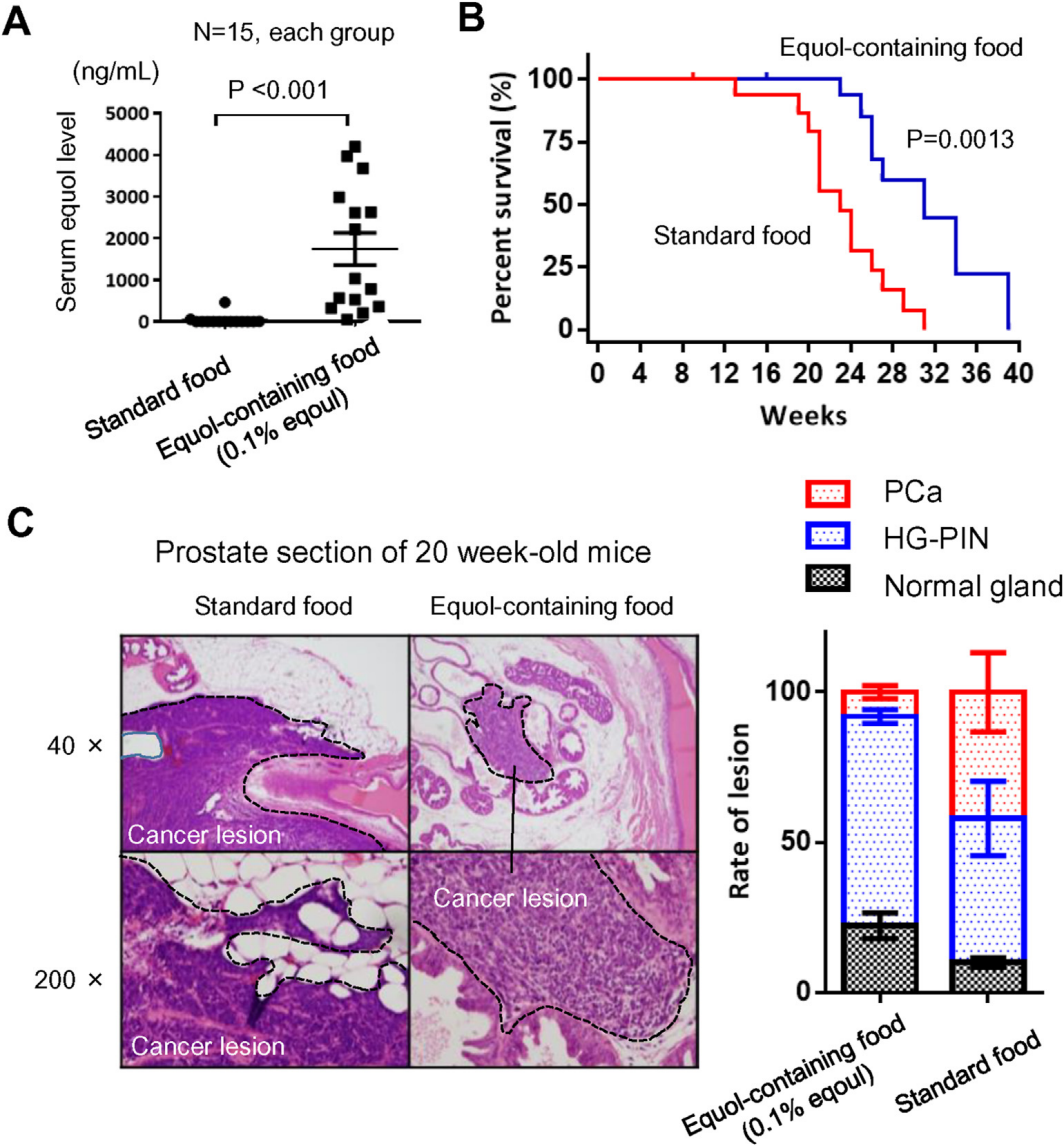
**Daily intake of equol prolongs survival and inhibits development of prostate cancer.** A) Serum level of equol in transgenic adenocarcinoma of the mouse prostate (TRAMP)/FVB mice comparing the equol-containing food group and the standard food group. A P-value is obtained using the Mann–Whitney U test. B) Cancer-specific survival curves of TRAMP/FVB mice comparing the equol-containing food group and the standard food group. A P-value is obtained using the log-rank test. C) Hematoxylin and eosin staining images of 20 week-old mice prostate sections comparing the equol-containing food group and the standard food group. Dashed black lines indicate borders between the cancer lesion and the normal area. Cancer occupation rate in the prostate section was calculated based on hematoxylin and eosin-stained sections and was compared between the equol-containing food group and the standard food group. PCa, prostate cancer; HG-PIN, high-grade prostatic intraepithelial neoplasia.

Our analysis included 76 patients diagnosed with organ-confined PCa between April 2008 and June 2011 (ethical approval ID: NMU-899). The materials and methods used are described in Supplementary File 1. In vitro experiments demonstrated that both daidzein and equol suppressed EMT and the migratory ability of PC-3 cells (Supplementary File 2). Immunostaining analysis using EMT markers, E-cadherin, vimentin, Slug, and Snail, revealed that higher Gleason scores were characterized by increased EMT potential (Fig. 2A). Pretreatment serum daidzein levels were associated with higher Gleason scores and higher rates of vimentin-positive cancer cells (Fig. 2B). Next, we performed an animal experiment using the orthotopic carcinogenesis mouse model, the transgenic adenocarcinoma of the mouse prostate model in the FVB background (transgenic adenocarcinoma of the mouse prostate/FVB hybrid mouse model).^71^ The mean serum level of equol was 1638 ng/mL in the equol-containing food group and 73 ng/mL in the standard food group (P < 0.001, Fig. 3A). The testosterone and DHT levels were not significantly different between the two groups (Supplementary File 3). Cancer-specific survival was significantly longer in the equol-containing food group than in the standard food group (31 weeks vs. 23 weeks, Fig. 3B). At 20 weeks of age, most mice developed high-grade prostatic intraepithelial neoplasia precancerous lesions, whereas cancer lesions were detected in some mice; the cancer occupancy rate was only 8% in the equol-containing food group and 42% in the standard food group (Fig. 3C). The tumor expression of E-cadherin was higher, and that of Slug was lower in the equol group than in the standard food group (data not shown). Moreover, the incidence of lung metastases was significantly lower compared with 27% in the equol group and 87% in the control group (P < 0.01). Our preclinical study suggests that treatment with daidzein and equol may be a potential chemopreventive approach to decrease the risk of PCa development and progression via the suppression of EMT. Our findings also provide evidence that isoflavones are potential agents for treating metastatic PCa.

## 5 Conclusion and future perspectives

A comprehensive understanding of the correlation between the urogenital microbiome and chronic prostatic inflammation could facilitate the development of novel strategies for PCa prevention. Further investigation would help to better understand the roles of gut microbiota dysbiosis in progression of prostate diseases and develop new therapeutic modalities.

## Funding source

The 10.13039/501100008879Japanese Foundation for Prostate Research Grant 2016, Japan (M.M.)

## Conflicts of interest

The authors disclose no potential conflicts of interest.
