# Supplementary material for: Prostate diseases and microbiome in the prostate, gut, and urine
Source: Prostate Int. 2022 Mar 29;10(2):96–107. doi: 10.1016/j.prnil.2022.03.004 (PMC9052083; doi:10.1016/j.prnil.2022.03.004)
Supplement: Multimedia component 1 [file mmc1.docx]

**MATERIALS AND METHODS**

***In-vitro assays***

Human PCa cell line, PC-3, were purchased from ATCC (Manassas, VA, USA) and maintained in RPMI-1640 growth medium (Nacalai Tesque Inc., Kyoto, Japan) supplemented with 10% FBS (ICN Biomedicals, Aurora, OH, USA), 100 U/mL penicillin, and 100 mg/mL streptomycin (Gibco, Grand Island, NY, USA) in a standard humidified incubator at 37°C in an atmosphere of 5% CO_2_. Recombinant human insulin-like growth factor 1 (IGF-1) and estradiol were purchased from R&D Systems (Minneapolis, MN). Daidzein and equol were purchased from LC Laboratories (Woburn, MA).

A migration assay was performed with the BD Falcon FluoroBlok Insert System (Becton Dickinson, Franklin Lakes, NJ, USA) according to the manufacturer’s directions. Briefly, cells were serum starved for 24 h before initiation of the assay. PC-3 cells were added to each insert at a density of 2.5 × 10^4^ cells/well in serum-free media with or without IGF-1, daidzein, equol, and estradiol. The lower chamber contained RPMI1640 media with 10% FBS as a chemoattractant. The cells were maintained in a humidified incubator with 5% CO2 at 37°C for 48 h. The cells attached to the bottom of the membrane were stained for 30 min with the cell viability indicator Calcein AM Fluorescent Dye (PromoKine, Heidelberg, Germany) and quantified using a microplate spectrophotometer (Infinite 200M PRO; Tecan, Männedorf, Switzerland) at 495 mm excitation and 515 nm emission. Cells were examined with a standard fluorescent microscopy.

***Immunohistochemical staining analysis of human prostate cancer tissue samples***

This study was approved by the Medical Ethics Committee of Nara Medical University, which waived the requirement for informed patient consent owing to the retrospective nature of the analysis (Ethical approval ID: NMU-899). A total of 76 patients who were diagnosed with organ-confined PCa between April 2008 and June 2011 in our hospital were included in this study. The clinicopathologic information and follow-up data were collected by retrospective chart review. All pathological examinations were generally performed under the guidance of two pathologists. Of 76 patients, 54 (71%) underwent radical prostatectomy with bilateral regional lymphadenectomy without any neoadjuvant therapies. Sixteen patients (21%) received androgen depletion therapy and the other six patients received radiation therapy. Immunohistochemical (IHC) staining of 54 RP specimens and 22 biopsy specimens using paraffin-embedded, formalin-fixed tissue blocks was performed as previously described [Miyake M et al. Neoplasia. 2017;19:250-251. doi: 10.1016/j.neo.2016.12.012]. Antibodies against E-cadherin (Cell Signaling Technology; clone 24E10, rabbit monoclonal #3195), vimentin (clone D21H3, rabbit monoclonal #5741, slug (clone C19G7, rabbit monoclonal #9585), and snail (clone C15D3, rabbit monoclonal #3879) were used as the primary antibodies (dilution 1:500 for all primary antibodies). The staining extent was scored based on the positive cell ratio using standard light microscopy. Staining outcomes were evaluated by two independent observers (Y. Tatsumi and T. Fuiji) who were blinded to the clinicopathological data of the 76 patients.

***The TRAMP/FVB hybrid mice***

Animal care was conducted in compliance with the recommendations of The Guide for Care and Use of Laboratory Animals (National Research Council). This study was approved by the animal facility Committee (protocol ID: 12126) and Committee for recombinant DNA experiments (protocol ID: 151) at Nara Medical University.

The transgenic adenocarcinoma of mouse prostate (TRAMP) mice (C57BL/6-Tg (TRAMP) 8247Ng/JXFVB/NJ)F1/J were purchased from Jackson Laboratories (JAX Mice and Services, Bar Harbor, ME, USA). We performed an experiment using the orthotopic carcinogenesis model mice, the TRAMP model in the FVB background. Male TRAMP mice heterozygous were generated by mating TRAMP+/− females (C57BL/6 background) with non-transgenic FVB males. In TRAMP mice, the minimal rat probasin (rPB) regulatory sequence targets SV40 early gene (T and t; Tag) expression speciﬁcally to prostatic epithelium. The rPB-SV40 T transgene was identified using DNA extracted from tail samples. Polymerase chain reaction was used to detect a 650-bp product using the following set of primers: 5’-GCGCTGCTGACTTTCTAAACATAAG-3’ (Pb-1, forward) and 5’-GAGCTCACGTTAAGTTTTGATGTGT-3’ (SV40Tag, reverse). The thermocycler was run for 35 cycles at 94°C (3 minutes), 94°C (30 seconds), 62°C (1 minutes), 72°C (3 minutes), and 4°C (end).

***Measurement of serum level of daidzein and equol***

Liquid chromatograph mass spectrometry (LC-MS) was utilized to measure the serum levels of the daidzein and equol. For a de-conjugation reaction, serum was treated with 2000 IU/mL of β-glucuronidase (Sigma-Aldrich Japan, Tokyo, Japan) for 24 h at 37°C, followed by mixture of serum and methanol of 4 double quantity. The mixture was filtered with a centrifugal separation filter (Centricut Ultramini W-MO, Kurabo Industries LTD, Japan) and subjected to LC-MS analyses. M Waters Micromass ZQ 4000 Mass Detector, Alliance HPLC System, and Photodiode Array (PDA) Detector (Nihon Waters K.K.) were used for measurement of the isoflavones. A CadenzaCD-C18 column (75 mm × 3.0 mm i.d, 3 μL Imtakt) was used as a separation column. A mixture of 0.1% (v/v) acetic acid in water and 100% acetonitrile was used as the mobile phase for isoflavone separation. Empower 2 Chromatography Data Software (Nihon Waters K.K.) was used for quantification.

***The effect of equol oral administration on TRAMP/FVB mice***

Four-week-old TRAMP/FVB hybrid mice were randomly divided into two groups: the equol-containing food group (n=30) and the standard food group (n=30). Four-week-old TRAMP/FVB hybrid mice were randomly into two groups: the equol-containing food group (n=30, a diet containing 0.1% equol) and the standard food group (n=30). We euthanize 5 animals in each group at 3 points (10 weeks, 20 weeks, 25 weeks), collect prostate, lung, liver, retroperitoneal lymph node tissues, perform hematoxylin and eosin staining, and cancer histological evaluation was performed on the occurrence and metastasis of the tumor. In addition, we examined the EMT regulation by evaluating the proliferation ability of cells with Ki-67 and immunohistochemical staining with EMT-related proteins.

The remaining 15 animals were reared until cancer death (10% or more weight loss or tumor palpation from the body surface), and the survival rate was compared by the log-rank test. Blood samples were taken from all mice and serum equol levels were measured using high performance liquid chromatography. Moreover, the testosterone level and dihydrosterone (DHT) level in blood were measured using ELISA kits (Abnova Corporation, Taipei, Taiwan).

***Statistical analysis***

Differences in migration were evaluated with Student-t test. The correlation of IHC staining intensity with Gleason score was assessed with Man-Whitney *U* test. Correlation between serum concentration of the isoflavons and tumor Gleason sum were analized with the Spearman rank-order correlation coefficient. IBM SPSS Version 21 (SPSS Inc., Chicago, IL) and PRISM software version 5.00 (San Diego, CA) were utilized for statistical analyses and plotting data, respectively. Statistical significance in this study was set at P < 0.05 and all reported *P* values were two-sided.
