# Supplementary figures and images for: Prostate diseases and microbiome in the prostate, gut, and urine
Source: Prostate Int. 2022 Mar 29;10(2):96–107. doi: 10.1016/j.prnil.2022.03.004 (PMC9052083; doi:10.1016/j.prnil.2022.03.004)

## Slide 1
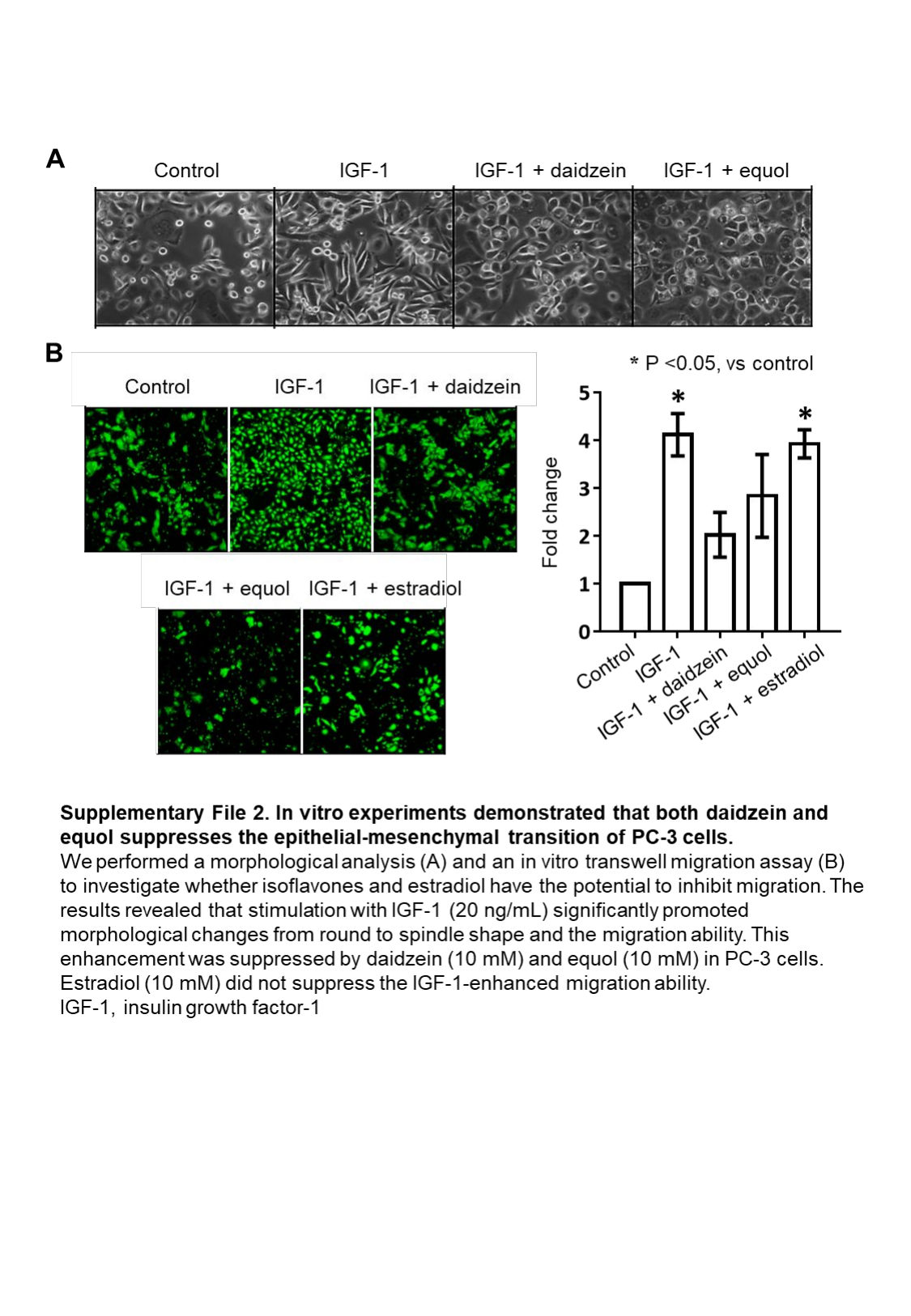

Supplement: Multimedia component 2 [file mmc2.pptx]

## Slide 1
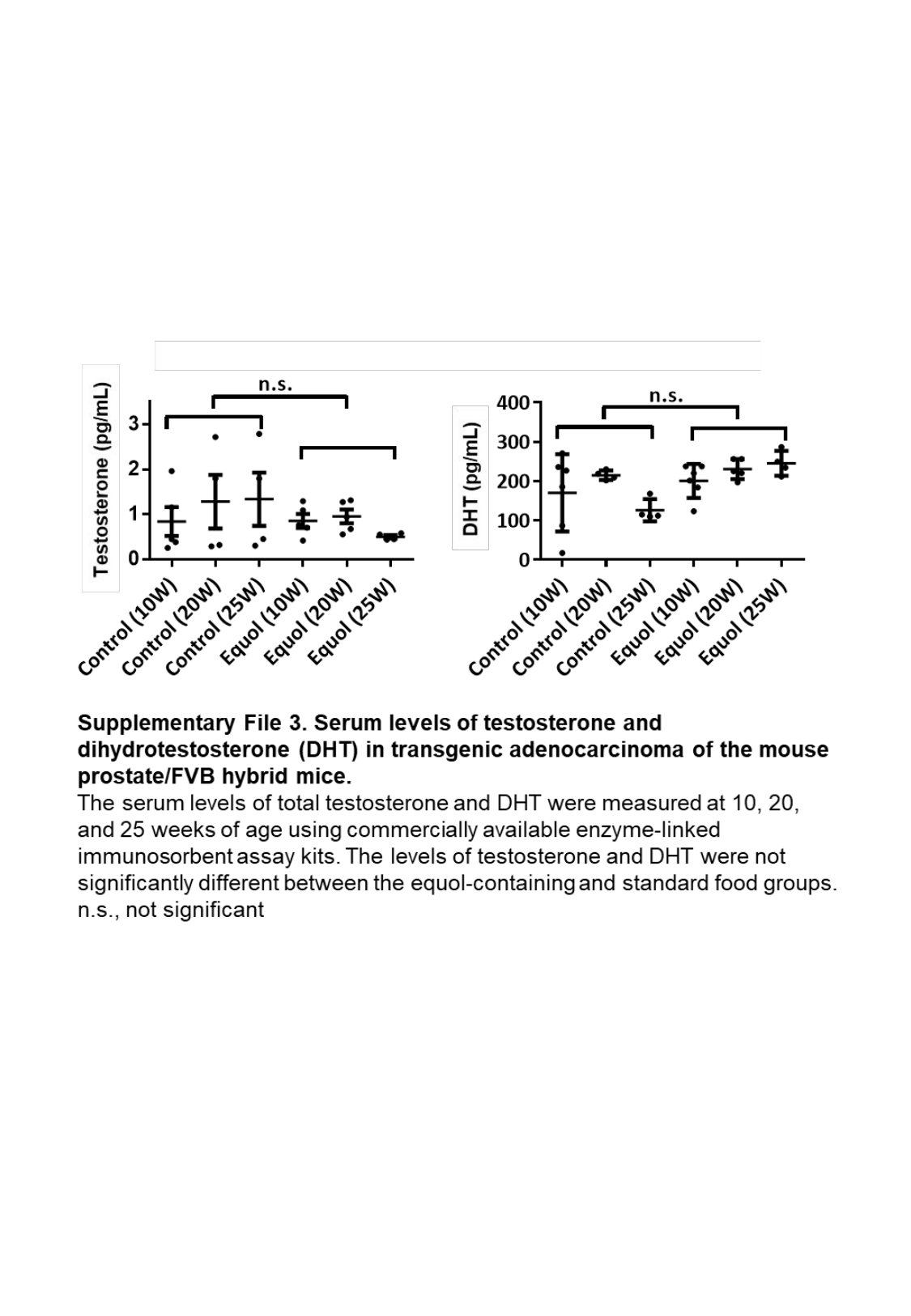

Supplement: Multimedia component 3 [file mmc3.pptx]
